# Serological and molecular detection of *Leptospira* spp. in ring-tailed coatis (*Nasua nasua*) from an Ecological Park

**DOI:** 10.1007/s11259-026-11372-4

**Published:** 2026-07-01

**Authors:** Gabriel Siqueira dos Santos, Denise Batista Nogueira Silva, Israel Barbosa Guedes, Rafael Rodrigues Soares, Nathália da Silveira Guimarães, Gisele Oliveira de Souza, Bruno Simões Sergio Petri, Haroldo Furuya, Ricardo Augusto Dias, José Soares Ferreira Neto, Marcos Bryan Heinemann

**Affiliations:** 1 Departamento de Medicina Veterinária Preventiva e Saúde Animal, Faculdade de Medicina Veterinária e Zootecnia, Universidade de São Paulo, Av. Prof. Orlando Marques de Paiva, 87 - Butantã, São Paulo, 05508-270 Brazil; 2 ARTEMIS Consultoria Ambiental, R. das Rosas, 126 - Mirandópolis, São Paulo, 04048-000 Brazil

**Keywords:** Leptospirosis, Zoonosis, Neglected diseases, Tropical diseases, Wild animals

## Abstract

Wild animals often play a role in the transmission of zoonotic pathogens. Among the wild mammals of South America, ring-tailed coatis (*Nasua nasua*) can present a high frequency of anti-*Leptospira* antibodies and carry *Leptospira* spp. This study aimed to evaluate the frequency of serum anti-*Leptospira* antibodies and perform the molecular identification of *Leptospira* spp. in ring-tailed coatis living in the Tietê River Ecological Park, in southeastern Brazil. Blood and urine samples were obtained from ring-tailed coatis. The serum was tested using the Microscopic Agglutination Test (MAT). Urine was cultured in semi-solid Ellinghausen–McCullough–Johnson–Harris medium. The inoculum was cultivated at 28 °C and monitored over six weeks. Total DNA from the blood and urine samples was extracted, and the 16 S rRNA gene and *lipL32* were searched by PCR. Of 192 animals, 36 (18.75%) had positive serum samples in the MAT. The most frequent serogroups in MAT were Grippotyphosa (11.46%; 22/192), Autumnalis (7.81%; 15/192) and Cynopteri (2.60%; 5/192). A total of 116 urine samples were obtained. None of the *Leptospira* spp. were isolated, twelve samples were positive for the 16 S rRNA gene (10.34%), and four were positive for the *lipL32* gene (3.4%). These results indicate a high frequency of serum anti-*Leptospira* spp. antibodies in coatis from the Tietê River Ecological Park and highlight the possibility of their potential role as carriers of pathogenic *Leptospira* spp. in urine.

## Introduction

Urbanization of the environment leads to the adaptation of wild animals to synanthropic behavior and increases the interaction of these species with humans and domestic animals. Synanthropic animals can act as transmitters of zoonotic diseases such as leptospirosis, an infectious illness caused by spirochetes of the genus *Leptospira* that frequently causes fever, acute renal and liver injuries, and death (Rajapakse 2022).

The transmission of *Leptospira* spp. not only occurs in direct contact with the urine of infected animals, but it can also occur indirectly in contaminated rivers, sewage, and soils (Bierque et al. 2020). Furthermore, tropical and subtropical environments, just like those in Southern Brazil, provide adequate conditions that allow the pathogen to maintain its animal-environment-human interface (Martins and Lilenbaum 2015).

Although rodents are the major urban reservoir of *Leptospira* spp., other synanthropic animals can harbor this pathogen and act as carriers (Fornazari et al. 2018; Padilha et al. 2021). However, because of the complexity of wildlife in Brazil, its epidemiological status regarding leptospirosis remains unclear. Previous studies revealed that wild carnivores in South America, including opossums (*Didelphis* spp.), grisons (*Galictis* spp.), and mainly coatis (*Nasua nasua*), have a high frequency of positive serological tests for *Leptospira* spp. (Vieira et al. 2018; Fornazari et al. 2018). In Brazil, however, the frequency of leptospirosis in synanthropic animals from South America remains unclear.

Ecological parks, in addition to their crucial role in conserving wildlife and native vegetation, also attract humans and domestic animals. This proximity can lead to incidents and direct or indirect transmission of *Leptospira* spp. (Mazzotta et al. 2023). Understanding the frequency of leptospirosis in synanthropic animals in ecological parks not only can reveal potential risks in zoonotic transmission of the pathogen but also raises points of concern in wildlife conservation issues and the sanitary status of the environment regarding zoonosis, creating an alert for public health.

## Materials and methods

### Study area and sampling

**Fig. 1:**
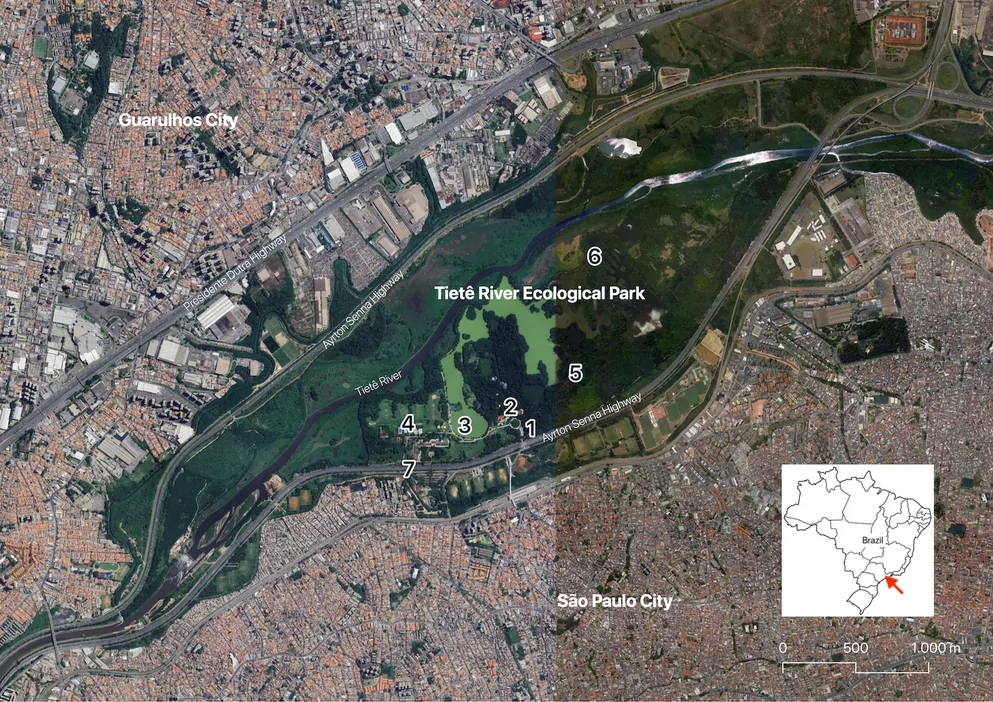
Map of Tietê River Ecological Park in the metropolitan region of São Paulo, Brazil. *located at latitude 23° 29’ 19” South and longitude 46° 31’ 14” West*: Numbers 1–7 indicate the capture locations of ring-tailed coatis (*Nasua nasua*)

The study was conducted in the Tietê River Ecological Park, in the region of the São Paulo and Guarulhos cities, Brazil (Fig. 1). The park covers approximately 1,400 hectares and is characterised by secondary Atlantic Forest vegetation. The area is composed of artificial lakes and ponds, forested areas, grasslands, beaches, mud, floodplain areas, swamps, and flooded fields. Even so, there are free-living animals, such as smooth-billed anis (*Crotophaga ani*), neotropic cormorants (*Nannopterum brasillianum*), caracaras (*Caracara plancus*), shiny cowbirds (*Molothrus bonariensis*), creamy-bellied thrushes (*Turdus amaurochalinus*), white-faced whistling ducks (*Dendrocygna viduata*), bees, white butterflies, tegus (*Tupinambis* spp.), and capybaras (*Hydrochoerus hydrochaeris*), besides ring-tailed coatis.

Between April and June 2023, during an animal population control programme in Tietê River Ecological Park involving the neutering of animals, blood and urine samples were obtained from ring-tailed coatis (*Nasua nasua*). Anaesthesia was performed using a combination of ketamine (10 mg/kg), midazolam (0.5 mg/kg), and xylazine (1 mg/kg). Forty minutes after administration, yohimbine (0.04 mL) was applied as an antagonist to reverse the effects of xylazine. Once the animal had been anaesthetised, 3 mL to 5 mL of blood samples were collected by venipuncture from the right jugular and, when possible, 3 mL to 5 mL of urine samples were obtained by cystocentesis from ring-tailed coatis (*Nasua nasua*) from the Tietê River Ecological Park and sent to the Bacterial Zoonosis Laboratory at the University of São Paulo. The blood was centrifuged for the extraction of the serum, and the material was frozen at -20 °C.

### Microscopic agglutination test and titration

The Microscopic Agglutination Test (MAT) was conducted following the methodologies outlined by Galton et al. (1965) and Cole et al. (1973). A panel of antigens representing 18 serogroups was utilied: Australis, Autumnalis, Ballum, Bataviae, Canicola, Celledoni, Cynopteri, Djasiman, Grippotyphosa, Hebdomadis, Icterohaemorrhagiae, Javanica, Panama, Pomona, Pyrogenes, Sejroe, Shermani, and Tarassovi.

Agglutination was observed using a dark-field microscope. Samples were deemed positive if at least 50% of the spirochetes were agglutinated in the 1:100 dilution (Adler and Moctezuma, 2010). Samples that tested reactive at the 1:100 dilution underwent serial dilution up to 1:6400, with the highest positive dilution value recorded as the titer of the sample. For each positive sample, the serogroup exhibiting the highest titers was considered the most likely infectious serogroup responsible for the infection (Guedes et al. 2021).

### *Leptospira* spp. culturing

Tubes containing semi-solid Ellinghausen McCullough Johnson Harris (EMJH) medium (Difco Laboratories, Franklin Lakes, NJ, USA) were seeded with 500 µL of urine, aiming for *Leptospira* spp. culturing and incubating at 28 °C. The tubes were observed every week for six weeks to check the superficial growth ring (Dinger’s zone). The presence of leptospires was confirmed by spirochete visualisation in dark-field microscopy.

### DNA extraction and PCR assay

The blood and urine samples were subjected to extraction and purification of the DNA with the PureLink^®^ Genomic DNA Mini Kit (Invitrogen, Thermo Fisher Scientific Inc., Carlsbad, CA, US). DNA amplification for *Leptospira* spp. was performed by classical PCR for the 16 S rRNA gene and *lipL32* gene according to the method described by Mérien et al. (1992) and Stoddard et al. (2009), respectively. Ultrapure water was used as a negative control, and a culture of *L. interrogans* serovar Copenhageni was used as a positive control.

### Data analysis

For statistical analysis, GraphPad Prism 9.0 (Version 9.0.0, GraphPad Software Inc., La Jolla, CA, USA) was utilised. Descriptive statistical analysis was conducted to determine the frequency of animals that tested positive in the MAT, as well as the frequency of serovars and serogroups identified in this test. Additionally, the frequency of samples positive for the screening of the 16 S rRNA and *lipL32* genes was assessed.

## Results and discussion

In this study, 192 ring-tailed coatis were captured across seven sites in Tietê River Ecological Park (see Fig. 1). Of these animals, 87 (45.3%) were males and 105 (54.7%) were females; regarding stage of li79 (41.1%) were classified as imature, 109 (56.8%) as adults, and 4 (2.1%) as elderly. Blood samples were obtained from all 192 animals, whereas urine samples were collected from 116 individuals. Consequently, 192 serum samples were analyzed by the Microscopic Agglutination Test (MAT), and all 192 blood samples together with the 116 urine samples were subjected to bacteriological culture and molecular detection assays.

Among the 192 animals, 36 [18.75% (CI 95%: 13.85% – 24.87%)] tested positive for at least one serogroup in the MAT. The most frequently identified serogroups were Grippotyphosa [11.46% (22/192)], Autumnalis [7.81% (15/192)], and Cynopteri [2.60% (5/192)]. These were followed by Australis [2.08% (4/192)], Icterohaemorrhagiae [2.08% (4/192)], Sejroe [1.56% (3/192)], Shermani [1.56% (3/192)], Hebdomadis [0.52% (1/192)], and Pyrogenes [0.52% (1/192)] (see Table 1).

**Table 1:** Seroprevalence and titers of anti-*Leptospira* spp. antibodies in ring-tailed coatis (*Nasua nasua*) from Tietê River Ecological Park

Serogroup	Serological titers								%reactives [IC 95%]
	100	200	400	800	1600	3200	6400	Total	
Australis	1	1	1	-	-	-	1	4	2.08% [0.81% – 5.24%]
Autumnalis	8	2	3	1	1	-	-	15	7.81% [4.78% – 12.49%]
Cynopteri	-	4	-	1	-	-	-	5	2.60% [1.11% – 5.95%]
Grippotyphosa	6	9	3	2	1	1	-	22	11.46% [7.67% – 16.77%]
Hebdomadis	-	-	1	-	-	-	-	1	0.52% [0.09% – 2.91%]
Icterohaemorrhagiae	1	1	1	-	1	-	-	4	2.08% [0.81% – 5.24%]
Pyrogenes	-	1	-	-	-	-	-	1	0.52% [0.09% – 2.91%]
Sejroe	1	1	-	-	1	-	-	3	1.56% [0.53% – 4.49%]
Shermani	1	-	1	1	-	-	-	3	1.56% [0.53% – 4.49%]

The wild animals studied in previous research also exhibited a significant frequency of antibodies against *Leptospira* (da Silva et al. 2023a, b; Fornazari et al. 2018). Given that all wild synanthropic animals in the Tietê River Ecological Park are free-range, they can interact with the coatis and influence the epidemiological context concerning these coatis. Notably, other screening studies have reported a high frequency of anti-Leptospira antibodies in coatis, surpassing that observed in other mammals in the same locations. Fornazari et al. (2018) conducted a study in a semi-rural area of São Paulo state and found a high prevalence of coatis testing positive in the Microscopic Agglutination Test (MAT) [26.8% (15/56)].

According to da Silva et al. 2023a, b; Vieira et al. (2018), the high frequency of *Leptospira* spp. in wild synanthropic carnivora may be due to their hunting behaviour and subsequent contact with infected prey, like synanthropic rodents that live in or near cities. Indeed, previous studies with other Carnivora species, including coatis, reported higher frequencies of seroreactivity against the Icterohaemorrhagiae serogroup. However, the animals from our study were less frequently seroreactive against this serogroup. Given that this serogroup is more common in urban synanthropic rodents, it is likely that these animals do not participate as transmitters in this specific epidemiologic situation.

The serogroups Grippotyphosa (*n* = 14) and Autumnalis (*n* = 5) also remained the most probable infectious serogroups, which resulted in the most notable titration serovar generator and were also the most frequent serogroups in the coatis (Table 2). This analysis eliminates possible cross-reactions with other serogroups, especially those serologically related (Guedes et al. 2021). Grippotyphosa and Autumnalis serogroups were reported in other screening in coatis, but at a lower frequency (Fornazari et al. 2018). On the other hand, in previous studies performed with coatis in a rural environment, the most prevalent serogroups were precisely those frequent in large domestic animals (Hardjo, Pomona, Australis, and Sejroe) and small synanthropic rodents (Icterohaemorrhagiae) (Fornazari et al. 2018).

**Table 2:** Most probable *Leptospira* spp. serogroup in ring-tailed coatis (*Nasua nasua*) from Tietê River Ecological Park

Animal	Most probable serogroup	Titration
3	Sejroe	1600
10	Shermani	400
13	Grippotyphosa	100
18	Hebdomadis	400
26	Grippotyphosa	100
31	Autumnalis	400
32	Autumnalis	100
43	Grippotyphosa	400
46	Grippotyphosa	100
47	Grippotyphosa	3200
56	Grippotyphosa	800
58	Grippotyphosa	100
67	Icterohaemorrhagiae	100
70	Autumnalis	100
88	Grippotyphosa	400
93	Icterohaemorrhagiae	1600
95	Autumnalis	400
105	Grippotyphosa	800
106	Grippotyphosa	200
107	Grippotyphosa	200
112	Grippotyphosa	200
115	Sejroe	100
120	Cynopteri	200
124	Sejroe	200
139	Grippotyphosa	200
140	Cynopteri	200
143	Grippotyphosa	200
148	Cynopteri	200
149	Pyrogenes	200
158	Australis	6400
163	Autumnalis	800
187	Cynopteri	800
198	Australis	100

The serogroups Grippothyphosa and Autumnalis have already been reported in other synanthropic mammals (da Silva et al. 2023a, b; Vieira et al. 2018), including in opossums and capybaras in the state of São Paulo, either by serology (Horta et al. 2016) or isolation (Moreno et al. 2016). Even though both serogroups are related to wild animals (Faine et al. 1999), these serogroups were previously isolated from asymptomatic cattle, which raises the hypothesis that leptospires may have the ability to adapt to the host, becoming a reservoir (Guedes et al. 2021). The serogroups Autumnalis and Icterohaemorrhagiae were the most frequently detected in dogs from the south region of São Paulo city by MAT, suggesting that these serogroups may be circulating in animals in the region (Santos et al. 2021).

The Ecological Tietê Park is bordered by two highways (Fig. 1) and traversed by the Tietê River. Once coatis do not exhibit aquatic habits in this park and are unable to cross the highways to access the surroundings, it is improbable that they could acquire *Leptospira* spp. strains from urbanised surroundings or contaminated water from the river. Furthermore, the Leptospira serogroups present in these coatis populations are likely to be Grippotyphosa and Autumnalis, both of which are uncommon in the typical urban synanthropic animal epidemiological cycle (Martins and Lilenbaum 2013).

In all 116 urine samples, twelve were positive for the 16 S rRNA gene (10.34%) and four were positive for the *lipL32* gene (3.4%). But, among the animals with urine positive for 16 S rRNA and those positive for *lipL32*, 25% (3/12 and 1/4, respectively*)* were positive in MAT.

Urine samples are frequently chosen due to the possibility of containing *Leptospira* spp. in the chronic phase of the disease. But, regarding leptospirosis screening in wild animals, capture, sedation, and the presence of urine in the bladder are simultaneous conditions for successful collection. The MAT negative results in animals positive for molecular detection of *Leptospira* spp. in urine may occur in chronic kidney disease in other mammal species (Santos et al. 2021; Soares et al. 2021).

None of the *Leptospira* spp. strains were isolated in the culturing procedure. Isolating *Leptospira* is always challenging, since the bacteria are fastidious. Furthermore, for successful isolation, the microorganism needs to be viable, which is not always achieved in urine samples, as metabolites and pH can compromise the viability of the agent (Faine et al. 1999). The lack of isolated strains and lower frequency molecular identification is an intrinsic challenge of *Leptospira* spp. screening and other studies with coatis and other mammals observed similar molecular detection frequencies (Vieira et al. 2018; Fornazari et al. 2018).

The molecular detection generally searches for genes universally present in bacteria, such as *gryB*, *rrs* (16 S rRNA gene), and *secY*, and *Leptospira*-specific membrane protein-encoding genes, such as *lipL21*, *lipL32*, *lipL41*, and *ligB* (Guernier et al. 2018). The 16 S rRNA gene was the first genetic marker used to identify *Leptospira* and remains a common target in many PCR diagnostic tests (Smythe et al. 2002). Although it presents a limited taxonomic resolution to differentiate *Leptospira* species within a clade, its application continues to be valued for initial screening. Furthermore, some recent studies still use it as a genetic target, underscoring its relevance (Di Azevedo and Lilenbaum 2021). The *lipL32* gene encodes an outer membrane lipoprotein initially identified as characteristic of pathogenic species and absent in non-pathogenic species (Stoddard et al. 2009). It is currently the most frequent target for *Leptospira* detection. However, despite its wide application, it is now known that it can also be present in intermediate strains (Picardeau 2017).

Many of the titers were 100 and 200 for both serogroups. In a previous study with wild mammals in Brazil, also most of the positive titration animals also presented low titers (Fornazari et al. 2018). Both findings could suggest contact with the agent or even highlight the role of these animals as maintenance hosts in wild environments (Fornazari et al. 2018).

The present study identified ring-tailed coatis (*Nasua nasua*) as possible reservoirs of *Leptospira*, contributing new insights into the epidemiology of this pathogen. A high frequency of anti-*Leptospira* antibodies and the presence of pathogenic *Leptospira* spp. in urine samples were observed in coatis from the Tietê River Ecological Park in São Paulo, Brazil. These findings highlight significant differences in infection frequency among species. Thus, the role of ring-tailed coatis as potential carriers of pathogenic *Leptospira* spp. underscores their importance in the maintenance and transmission of this pathogen in the studied environment.

## Data Availability

No datasets were generated or analysed during the current study.
